# Larval exposure to the neonicotinoid imidacloprid impacts adult size in the farmland butterfly* Pieris brassicae*

**DOI:** 10.7717/peerj.4772

**Published:** 2018-05-18

**Authors:** Penelope R. Whitehorn, George Norville, Andre Gilburn, Dave Goulson

**Affiliations:** 1 Biological and Environmental Sciences, Faculty of Natural Sciences, The University of Stirling, Stirling, United Kingdom; 2 Institute of Meteorology and Climate Research–Atmospheric Environmental Research (IMK-IFU), Karlsruhe Institute of Technology, Garmisch-Partenkirchen, Germany; 3 School of Life Sciences, University of Sussex, Brighton, United Kingdom

**Keywords:** Pesticides, Neonicotinoids, Butterflies, Non-target

## Abstract

Populations of farmland butterflies have been suffering from substantial population declines in recent decades. These declines have been correlated with neonicotinoid usage both in Europe and North America but experimental evidence linking these correlations is lacking. The potential for non-target butterflies to be exposed to trace levels of neonicotinoids is high, due to the widespread contamination of agricultural soils and wild plants in field margins. Here we provide experimental evidence that field realistic, sub-lethal exposure to the neonicotinoid imidacloprid negatively impacts the development of the common farmland butterfly *Pieris brassicae*. Cabbage plants were watered with either 0, 1, 10, 100 or 200 parts per billion imidacloprid, to represent field margin plants growing in contaminated agricultural soils and these were fed to *P. brassicae* larvae. The approximate digestibility (AD) of the cabbage as well as behavioural responses by the larvae to simulated predator attacks were measured but neither were affected by neonicotinoid treatment. However, the duration of pupation and the size of the adult butterflies were both significantly reduced in the exposed butterflies compared to the controls, suggesting that adult fitness is compromised through exposure to this neonicotinoid.

## Introduction

The use of neonicotinoids has been implicated in the global decline of pollinators and this has caused much debate and controversy (Goulson, 2013; Stokstad, 2013). Since their introduction in the early 1990s they have become the most widely used insecticides in the world. Their success is partly due to their systemic nature, which allows them to be applied in a variety of ways, such as seed dressings, foliar sprays and soil drenches (Jeschke et al., 2011). After application, the chemicals are able to spread throughout the plant tissues, hence protecting the whole plant from pests.

However, the impact of neonicotinoids extends beyond the crop plants and the target pest species and this has brought their widespread use into question (Goulson, 2013; Van der Sluijs et al., 2015). For example, beneficial insects can be exposed to these insecticides when they forage on flowering crops, as trace levels have been detected in the pollen and nectar of treated plants (Blacquière et al., 2012; David et al., 2016). Non-target invertebrates are also exposed to neonicotinoids when they forage on non-crop plants growing in agricultural lands (Krupke et al., 2012; Botías et al., 2016). Such contamination of non-target plants can occur from the dust produced during the drilling of treated seeds, which drifts onto surrounding vegetation and has been shown to contain high concentrations of neonicotinoids (Krupke et al., 2012; Girolami et al., 2013; Limay-Rios et al., 2016).

A debatably more serious route of contamination is through the soil; when used as seed dressings, only 1.6 to 20% of the active ingredient of neonicotinoids is taken up by the crop and the remainder stays in the soil (Sur & Stork, 2003). The half-lives of neonicotinoids in soils can exceed 1,000 days and so they can accumulate when used repeatedly (Bonmatin et al., 2015). This persistence, combined with a high potential for lateral movement and leaching, can cause widespread contamination of agricultural areas (Sánchez-Bayo et al., 2007; Gupta, Gajbhiye & Gupta, 2008; Kurwadkar et al., 2014). A study of conventionally managed farmland in France found that 65% of soil samples contained >1 ppb imidacloprid (despite only 15% of sites having been planted with treated seeds in the same year), 14% of samples contained between 10 and 100 ppb and 4% contained over 100 ppb (Bonmatin et al., 2005). In a study in Eastern England, clothianidin, thiamethoxam and imidacloprid were all detected in fields where no treatment had been applied in the previous three years—imidacloprid at levels up to 10.7 ppb (Jones, Harrington & Turnbull, 2014). Botías et al. (2015) sampled agricultural soils and found both the soil directly beneath the treated crop and also the soils in the field margins to be highly contaminated with neonicotinoids, again even when none of the detected chemical had been applied in the previous three years. This soil contamination can lead to uptake into field margin plants and, indeed, neonicotinoid residues have been found in 52% of foliage samples collected from wild plants in oil seed rape field margins (Botías et al., 2016). This demonstrates that non-target insects in field margins are likely to be chronically exposed to highly variable concentrations of neonicotinoids in plant tissues.

To date, much of the work on the effect of neonicotinoids on non-target invertebrates has focused on honeybees and species of bumblebee. Exposure to field realistic concentrations has been shown to have substantial sub-lethal effects, such as reduced foraging efficiency (Gill, Ramos-Rodriguez & Raine, 2012; Feltham, Park & Goulson, 2014), impaired navigation (Henry et al., 2012), a reduction in learning and memory (Stanley, Smith & Raine, 2015), reduced ovary development (Baron, Raine & Brown, 2017) and reduced reproductive success (Whitehorn et al., 2012; Baron et al., 2017b). Such sub-lethal effects can have substantial negative impacts at the population level (Rundlöf et al., 2015; Woodcock et al., 2016) and neonicotinoids have been implicated as a contributory factor in the global decline of pollinators (Van der Sluijs et al., 2015). However, little research has investigated the impact on other non-target invertebrate taxa, such as butterflies (Pisa et al., 2015). Farmland butterflies in England declined by 56% between 2002 and 2009, despite large investments in the management of areas for biodiversity conservation during this time (Brereton et al., 2011). The declines have also occurred despite predictions that moderate climate change will benefit UK butterflies due to warmer summer temperatures (Roy et al., 2001).

The possibility that the recent declines in butterfly numbers may have been driven by the increasing usage of neonicotinoids has been modelled by Gilburn et al. (2015). This paper extended previous models of the UK population indices of 17 species of widespread farmland butterflies (Roy et al., 2001) with the additional explanatory variable of neonicotinoid pesticides usage. These models found a strong negative association between 15 of the 17 butterfly species population indices and the number of hectares of farmland treated with neonicotinoids the previous year. These population declines have also primarily occurred in England where neonicotinoid usage is at its highest; in contrast in Scotland, where neonicotinoid usage is much lower, butterfly numbers are stable. Similar negative associations between butterfly populations and neonicotinoid application have been observed in California, particularly after 1997 when imidacloprid started to be used in this state (Forister et al., 2016). Despite these compelling correlations, there is little experimental evidence demonstrating the effects of trace levels of neonicotinoids on non-target butterfly species. It has been found that doses as low as 1 ppb of clothianidin have sub-lethal impacts on Monach butterflies (*Danaus plexippus*) and contamination of the larval foodplant, milkweek, may be contributing to the mortality of neonates (Pecenka & Lundgren, 2015). In this paper we seek to further address this knowledge gap by investigating the sub-lethal effects of the neonicotinoid imidacloprid on the large white butterfly (*Pieris brassicae*). This butterfly is a widespread species and its larvae feed on wild or cultivated species of the Brassicaceae family (Feltwell, 1982). It was chosen as a study species due to ease of handling in laboratory and because it currently has a stable population in the UK, but it is declining at some sites, particularly in the more agricultural south and east of the country (UKBMS), where neonicotinoid usage is high.

## Materials & Methods

Cabbage plants (*Brassica oleracea* var. *capitata* L.) were grown from seed purchased from an organic supplier (Laura’s Organics Ltd., Wigan, UK). Pure imidacloprid (Sigma-Aldrich, UK) was dissolved in a known volume of distilled water and used to dose the cabbage plants. Each time the plants were watered they received either 0 (control), 1, 10, 100 or 200 parts per billion (ppb) imidacloprid to create five treatment groups of cabbage plants. The treated water was added directly to the soil beneath the plants to avoid additional residue contamination on the leaves. These levels were chosen to fall within the range of concentrations of neonicotinoids that have been found in agricultural soils (Bonmatin et al., 2005; Botías et al., 2015). It is known that imidacloprid has a high xylem mobility through cabbage plants (Buchholz & Nauen, 2002) and the cabbage plants therefore represented agricultural field margin vegetation grown in contaminated conditions.

*Pieris brassicae* eggs were sourced from a butterfly breeder (M. Hoare, Knoles butterflies) and consisted of one sibling group, originating from one female. When the larvae hatched they were split into five treatment groups and placed in ventilated plastic boxes in a controlled environment cabinet under a 16 h L/ 8 h D regime at 20°C (see Table 1 for sample sizes). Each of the five boxes were provided with cabbage leaves from one of the imidacloprid treatment groups *ad libitum*. When the majority of the caterpillars were heavier than 30 mg (range of initial mass 19–124 mg, mean 55.7 mg), they were weighed and placed individually in boxes. Solitary conditions have been previously shown to not impact the development of this normally gregarious species (Higginson et al., 2011). The solitary caterpillars continued to feed on the cabbage treatment they had originally received and this was refreshed three times, every third or fourth day. The weight of cabbage placed into each box, the weight of cabbage removed and the weight of frass produced was recorded each time, allowing the ‘Approximate Digestibility (AD)’ to be calculated (Waldbauer, 1968) at the three time points.

**Table 1:** Sample sizes across the treatments and larval survival.

Neonic treatment	Sample size	Predator treatment	Sample size	No. survived to adulthood
Control	32	P	15	14
		NP	17	14
1 ppb	31	P	15	14
		NP	16	15
10 ppb	32	P	15	14
		NP	17	13
100 ppb	32	P	15	15
		NP	17	15
200 ppb	31	P	15	13
		NP	16	16

\begin{eqnarray*}\text{AD}=\text{Food}~\text{ingested}-\text{Frass}~\text{produced}/\text{Food}~\text{ingested}. \end{eqnarray*}

In order to investigate whether any impacts of neonicotinoids are amplified by additional stress, the individually housed caterpillars were then randomly allocated to a predator attack treatment. Half of each cabbage treatment group were subjected to a simulated predator attack, and the other half were left undisturbed. We simulated a predator attack by lightly pinching the caterpillars’ abdomen three times, using soft forceps (Storkbill fine blunt forceps; NHBS, Totnes, UK). The caterpillar’s immediate response to the attack was recorded, including whether it regurgitated, moved its head or tail or dropped from the leaf. These behaviours were not mutually exclusive and caterpillars often displayed more than one response. The predator attacks occurred a total of three times and were carried out once every second or third day. The date that the caterpillar pupated was recorded and the pupae were allowed to eclose in the individual boxes.

Once the butterflies had eclosed, they were transferred to flight cages measuring 60 × 40 × 60 cm (width × depth × height), located in an unheated polytunnel on the Stirling University campus. Butterflies were individually marked on their hind wings and kept in their treatment groups, meaning there were a total of ten flight cages (1 ppb predator; 1 ppb no predator; 10 ppb predator; 10 ppb no predator; 100 ppb predator; 100 ppb no predator; 200 ppb predator; 200 ppb no predator; control predator; control no predator; Table 1). After death of the adults the forewings of each individual were measured, a standard indicator of overall size in butterflies (Gage, 1994).

## Statistical Analysis

Data were analysed in R, version 3.4.1 (R Core Team, 2017). Binomial generalized linear mixed effect models were used to analyse determinants of the Approximate Digestibility (AD) of the cabbage leaves by the larvae. Neonicotinoid treatment (0, 1, 10, 100, 200 ppb) and predator treatment (Predator (P) or no predator (NP)) were entered as factors, the initial larval weight and the time point (1, 2, 3) were entered as co-variates and the individual larval ID was entered as a random effect. Binomial generalized linear mixed effect models were also used to analyse the behaviour of the subset of larvae that underwent simulated predator attacks. Separate (binomial glmer) models analysed the probabilities of larvae reacting, regurgitating, moving their head, moving their tail and, finally, dropping from the leaf. Additionally, a generalized linear mixed effect model with Poisson distribution was used to analyse the total number of behaviours performed. In all behaviour models the neonicotinoid treatment (0, 1, 10, 100, 200 ppb) was entered as a factor, the initial larval weight and the time point (1, 2, 3) were entered as co-variates and the individual larval ID was entered as a random effect. In all models, fit was assessed using chi-square tests on the log-likelihood values to compare different models. Visual inspection of residual plots did not reveal any deviations from normality.

Pupation length, measured in days, was compared across firstly the neonicotinoid treatment groups and secondly across the predator treatment groups with Kruskall-Wallis tests, due to its non-normal distribution. Post hoc Wilcoxon tests were used to test pairwise comparisons and these were Bonferroni corrected for multiple comparisons. Finally, adult forewing length was analysed with a general linear model, with neonicotinoid treatment, predator treatment and sex entered as factors and the initial larval weight as a co-variate. Visual inspection of residual plots did not reveal any deviations from normality.

## Results

A total of 158 larvae were split across the five neonicotinoid treatment groups and 143 of these survived until adulthood (mortality was less than 10% of the initial number of larvae), with no difference among treatments in larval survival (Table 1).

### Approximate Digestibility (AD)

The mean Approximate Digestibility (AD) across all larvae was 51.95% (range 38.37 –81.23%). Approximate Digestibility was not affected by either the neonicotinoid treatment, the simulated predator treatment or initial weight of the larvae (*χ*^2^ = 3.87, *df* = 4, *p* = 0.424; *χ*^2^ = 0.964, *df* = 1, *P* = 0.326 and *χ*^2^ = 0.293, *df* = 1, *P* = 0.588 respectively). However, AD declined significantly over time (*χ*^2^ = 21.63, *df* = 1, *P* < 0.001) (Table 2).

**Figure 1:**
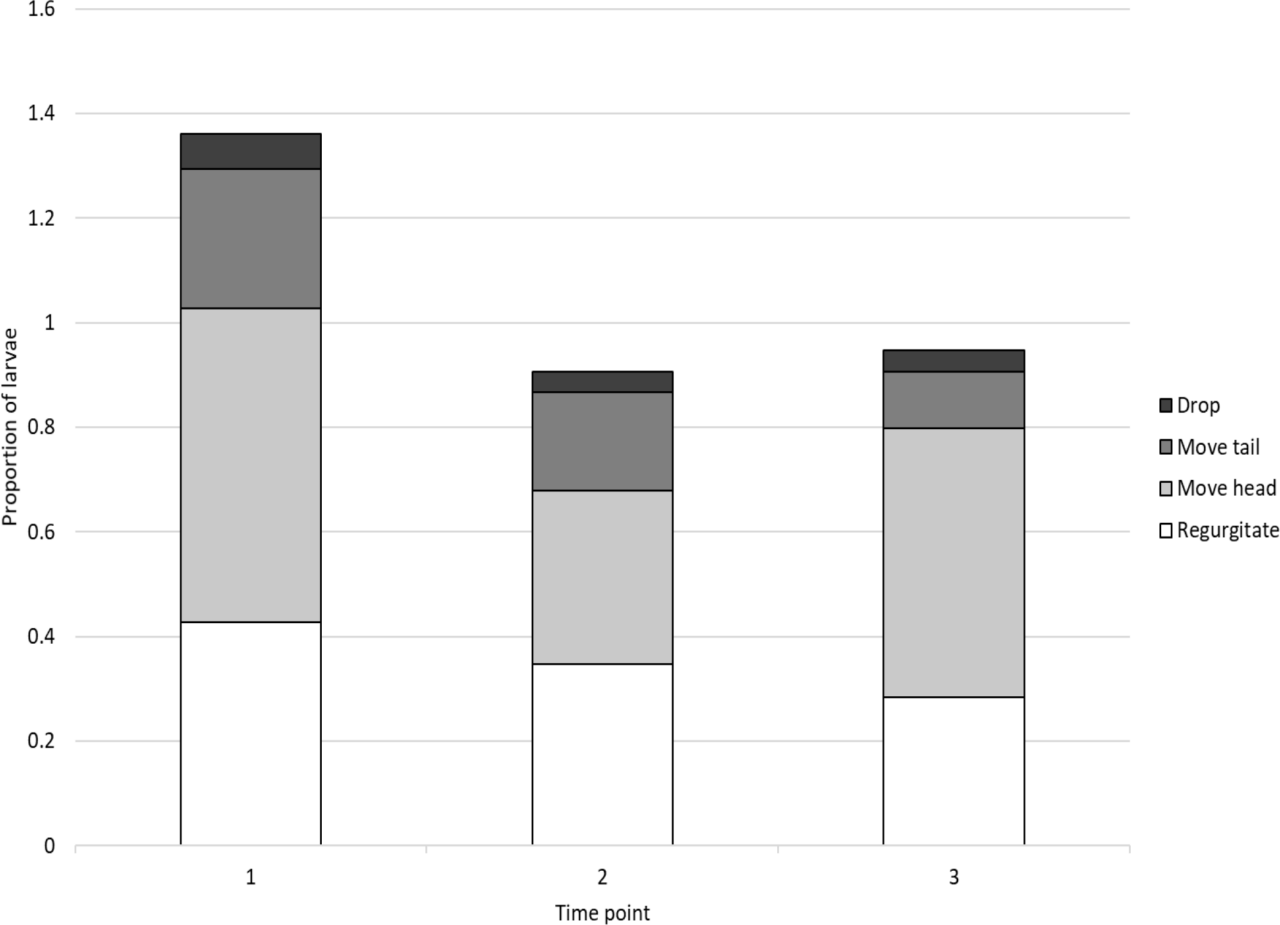
The proportion of larvae reacting with the four behaviours at each time point.

### Anti-predator behaviour

More than half (58.5%) of larvae reacted when subjected to a simulated predator attack but the probability of a larvae reacting or not was not affected by neonicotinoid treatment, initial larval weight or the time point (*χ*^2^ = 2.65, *p* = 0.617; *χ*^2^ = 0.730, *P* = 0.393 and *χ*^2^ = 0.556, *P* = 0.456 respectively). The most common responses were head movements and regurgitation (48.2% and 35.3% reactions, respectively). Tail movements and dropping from the leaf were less commonly observed (18.8% and 4.9% reactions). Behaviours were not mutually exclusive and larvae often displayed more than one response (see Fig. 1 for the breakdown of behaviours at the three time points).

**Table 2:** Parameter estimates and 95% CIs from the generalised linear mixed effect models for Approximate Digestibility (AD). The parameter estimates shown here are with reference to the control neonicotinoid treatment and the non-predator treatment group.

		Parameter estimate	Lower 95% CI	Upper 95% CI
Intercept		1.052	0.346	1.758
Neonic Treatment	1 ppb	−0.124	−0.584	0.336
	10 ppb	0.116	−0.337	0.569
	100 ppb	0.302	−0.152	0.755
	200 ppb	−0.048	−0.493	0.397
Predator treatment	P	0.144	−0.138	0.425
Initial larval weight		1.592	−4.173	7.356
Time		**−0.506**	**−0.726**	**−6.285**

The neonicotinoid treatment had no impact on the number of total behaviours performed (*χ*^2^ = 2.18, *df* = 4, *p* = 0.703) or on the probability of a larvae responding with a particular behaviour (regurgitation: *χ*^2^ = 1.64, *df* = 4, *p* = 0.802; move head: *χ*^2^ = 2.30, *df* = 4, *p* = 0.681; move tail: *χ*^2^ = 2.40, *df* = 4, *p* = 0.662; drop: *χ*^2^ = 2.36, *df* = 4, *p* = 0.669, see Table 3 for parameter estimates and 95% CIs from the generalised linear mixed effect models). However, the number of total behaviours performed declined over time and decreased with increasing initial larval weight (*χ*^2^ = 6.42, *df* = 1, *p* = 0.011 and *χ*^2^ = 4.74, *df* = 1, *p* = 0.029 respectively, Table 3). No variable predicted the probability of larvae moving their head or dropping from the leaf (Table 3) but the probability of a larvae regurgitating was lower for larvae that had a greater initial weight (*χ*^2^ = 6.19, *df* = 1, *p* = 0.013, Table 3) and the probability of responding with a tail movement declined over time (*χ*^2^ = 6.31, *df* = 1, *p* = 0.012, Table 3).

**Table 3:** Parameter estimates and 95% CIs from the generalised linear mixed effect models for the behavioural responses to simulated predator attack. The parameter estimates shown here are with reference to the control neonicotinoid treatment.

		**Probability of reacting**			**Regurgitation**			**Move head**			**Move tail**			**Drop**			**Total behaviours performed**		
		Parameter estimate	Lower 95% CI	Upper 95% CI	Parameter estimate	Lower 95% CI	Upper 95% CI	Parameter estimate	Lower 95% CI	Upper 95% CI	Parameter estimate	Lower 95% CI	Upper 95% CI	Parameter estimate	Lower 95% CI	Upper 95% CI	Parameter estimate	Lower 95% CI	Upper 95% CI
Intercept		0.681	−0.401	1.762	0.867	−0.336	2.070	0.448	0.162	0.734	0.200	−1.099	1.499	−0.854	−3.122	1.414	0.735	0.210	1.260
Neonic Treatment	1 ppb	0.005	−0.883	0.893	−0.328	−1.357	0.701	−0.273	−1.123	0.578	−0.410	−1.566	0.746	−0.950	−3.273	1.373	−0.209	−0.692	0.273
	10 ppb	0.183	−0.707	1.073	−0.007	−2.039	2.024	0.175	−0.666	1.015	−0.430	−1.583	0.723	−0.277	−2.138	1.584	−0.014	−0.473	0.445
	100 ppb	0.651	−0.264	1.567	0.329	−0.651	1.309	0.333	−0.505	1.171	0.156	−0.881	1.193	−1.116	−3.431	1.199	0.131	−0.311	0.572
	200 ppb	0.141	−0.743	1.025	0.066	−0.920	1.051	−0.038	−0.873	0.798	0.244	−0.778	1.266	0.220	−1.365	1.806	0.073	−0.372	0.517
Initial larval weight		−4.698	−16.01	6.609	**−15.582**	**−29.07**	**−2.10**	−3.673	−14.33	6.983	−10.61	−25.10	3.89	−29.464	−60.65	1.723	**−6.758**	**−12.77**	**−0.748**
Time		−0.128	−0.461	0.461	−0.350	−0.712	0.013	−0.178	−0.503	0.147	**−0.550**	**−0.989**	**−0.112**	−0.287	−1.051	0.478	**−0.201**	**−0.357**	**−0.046**

### Pupation

**Figure 2:**
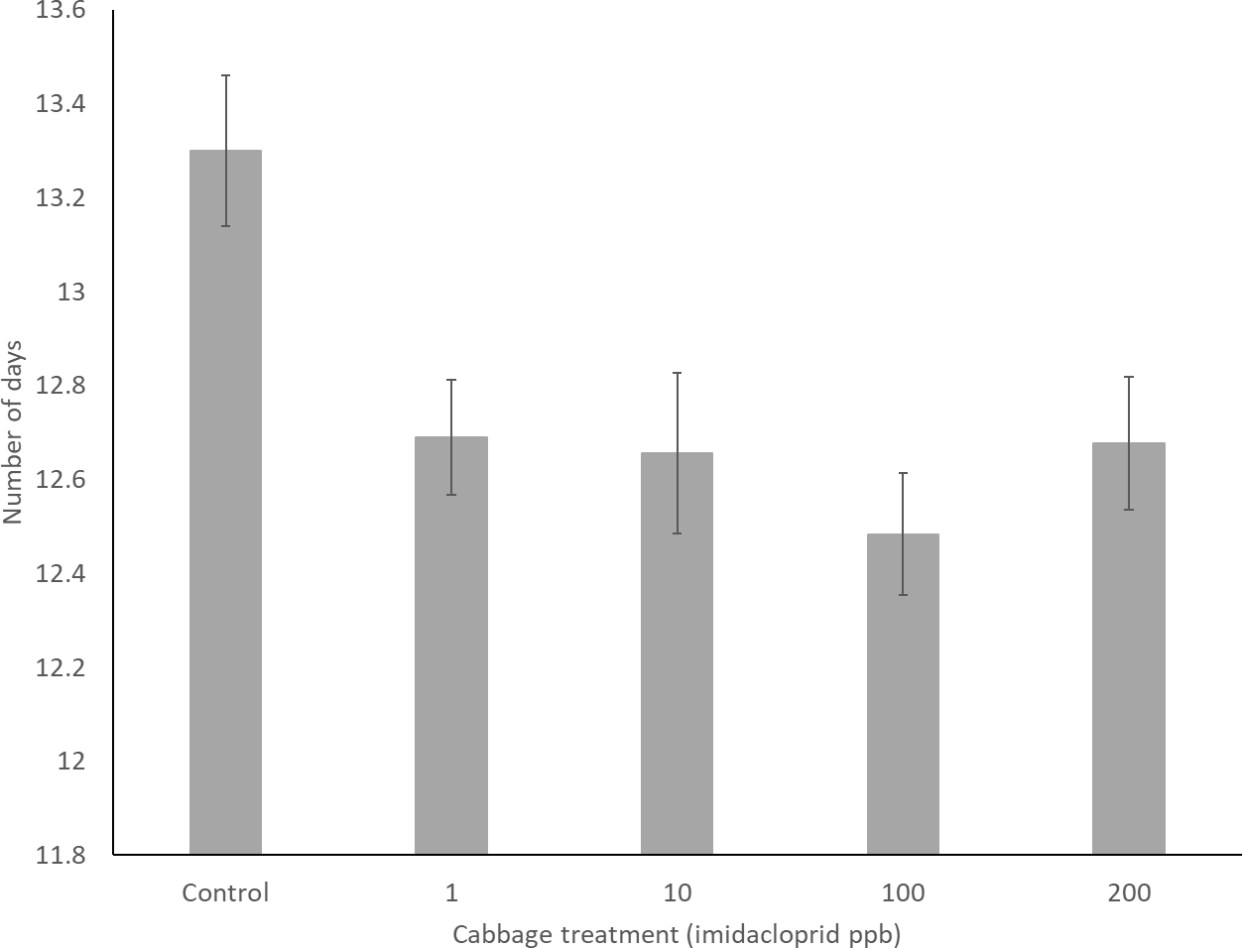
Pupation duration in days in the five cabbage treatment groups.

The mean duration of pupation was 12.76 (±0.145) days and this was impacted by neonicotinoid treatment (*H*(4) = 21.88, *p* < 0.001) with the control larvae pupating for a significantly longer time than any of the treated groups (Fig. 2). Pupation length was not impacted by predator treatment (H (1) = 0.137, *p* = 0.712).

### Adult forewing length

**Figure 3:**
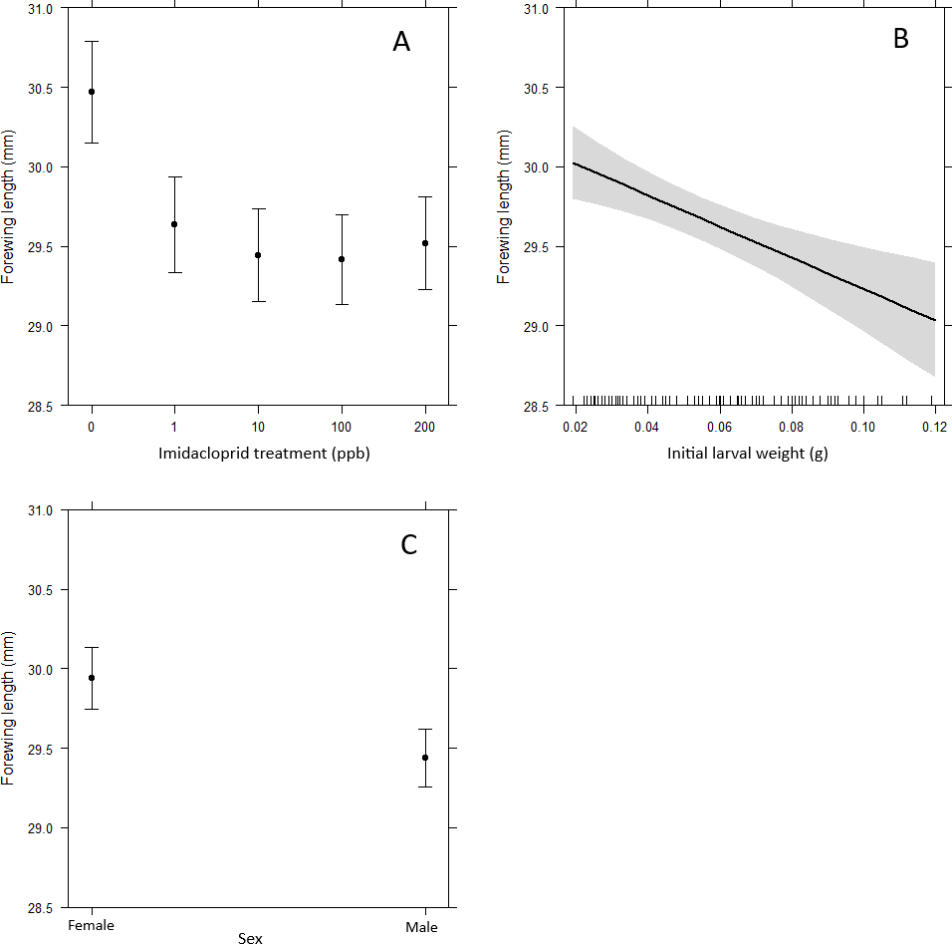
Variables predicting the forewing length of adult butterflies. (A) Adult forewing length was significantly greater in the control butterflies compared to those exposed to imidacloprid (compared to control: 1ppb*t*_(4)_ =  − 3.74, *p* < 0.001; 10 ppb *t*_(4)_ =  − 4.71, *p* < 0.001; 100 ppb *t*_(4)_ =  − 4.94, *p* < 0.001; 200  ppb *t*_(4)_ =  − 4.36, *p* < 0.001). (B) Initial larval weight had a significant negative correlation with adult forewing length (*t*_(1)_ =  − 3.45, *p* < 0.001). (C) Adult females had significantly larger forewings than adult males (*t*_(1)_ =  − 3.73, *p* < 0.001).

Butterflies in the neonicotinoid treatment groups had significantly smaller forewings compared to control butterflies (1 ppb, *t*(4) =  − 3.74, *p* < 0.001; 10 ppb, *t*(4) =  − 4.71, *p* < 0.001; 100 ppb, *t*(4) =  − 4.94, *p* < 0.001; 200 ppb, *t*(4) =  − 4.36, *p* < 0.001, Fig. 3A). Additionally, male butterflies had smaller forewings than females, as expected for this species (*t*(1) =  − 3.73, *p* < 0.001, Fig. 3C) and forewing length declined with increasing initial larval weight (*t*(1) =  − 3.45, *p* < 0.001, Fig. 3B). The predator treatment had no impact on the forewing length of adults (*t*(1) =  − 1.21, *p* = 0.228).

## Discussion

We have shown that when larvae of the common farmland butterfly *Pieris brassicae* are exposed to sub-lethal doses of the neonicotinoid imidacloprid, they have a shorter pupation period and the adult butterflies emerge significantly smaller. This effect was observed across all neonicotinoid doses, with exposure from 1 ppb up to 200 ppb having the same impact (Fig. 3). In Lepidoptera, there is a strong positive relationship between body size and fitness, with larger individuals having greater reproductive success (Bauerfeind & Fischer, 2008; Higginson et al., 2011), as well as a better flight ability and endurance (Shirai, 1995). Therefore, the results of this study provide support for the hypothesis that the recent negative population trends observed in farmland butterflies might be a consequence of increasing neonicotinoid usage (Gilburn et al., 2015; Forister et al., 2016).

The neonicotinoid treatment did not appear to affect the assimilation of nutrients from the cabbage leaves by the larvae as the approximate digestibility (AD) was not different among the treatments. AD did, however, decline with the age of the larvae, which is expected as smaller larvae tend to select the parts of the leaf they eat, avoiding the less digestible leaf veins, whilst larger larvae are less discriminating (Panizzi & Parra, 2012). It is possible that the neonicotinoid impacted the efficiency of conversion of the digested food into biomass and it would be interesting to explore this mechanism in a future study.

Our study found that it is the adult stage of this species that is impacted by neonicotinoid exposure as a larva and it would be worthwhile to further investigate the impacts of these chemicals on the behaviour and reproductive success of adults. Neonicotinoids impact the ability of bees to effectively navigate and forage (e.g., Henry et al., 2012; Feltham, Park & Goulson, 2014) and it is possible that similar impacts in butterflies would decrease their ability to find suitable forage flowers and hence impact their survival in the field. Additionally, as *P. brassicae* is a migratory species, small impacts on navigation could have profound impacts when moving over hundreds of kilometres. Sub-lethal doses of neonicotinoids are also known to impact the reproductive success of bumblebees (e.g., Whitehorn et al., 2012; Baron, Raine & Brown, 2017), and a similar impact on the fecundity of adult butterflies would explain the dramatic population declines that have been observed in agricultural regions. It has been found that imidacloprid can reduce the development time of pupae as well as the fecundity and survival of the pest moth species *Helicoverpa armigera* (Ahmad, Ansari & Ahmad, 2013), so it would be beneficial to investigate whether these impacts extend to non-target Lepidoptera species, where such effects would not be advantageous.

Interestingly, the simulated predator attacks had no impact on either larval development or adult body size of *P brassicae* in this experiment. This is contrary to the findings of Higginson et al. (2011), who found that there was a growth and survival cost to predator attacks in *P. brassicae*. However, their study induced the larvae to defensively regurgitate in each simulated predator attack and so the later costs were a consequence of the regurgitation (through loss of body fluids and nutrients), rather than the attack itself. Although regurgitation was a commonly observed defensive behaviour in our experiment, it comprised only 35% of the observed reactions and therefore possibly did not occur frequently enough to cause observable negative effects on the developing larvae. We did observe that the larvae that had a greater initial weight were less likely to regurgitate and performed fewer defensive behaviours overall. This could be linked to the trade-offs between growth and defensive behaviours, with the larger larvae on a pathway to optimise growth at the expense of anti-predator strategies (Higginson et al., 2011). We also observed that the amount of behaviours performed and specifically the propensity to move their tails, declined over time as the larvae aged. This could be linked to the fact that the larvae became less active as they prepared to pupate (P Whitehorn, pers. obs., 2014).

## Conclusions

In conclusion, this study provides some initial experimental evidence for the sub-lethal impacts of neonicotinoids on a common butterfly species. We find that exposure to levels as low as 1 ppb have negative consequences for the size of adult butterflies, a strong indicator of fitness in butterflies. Further research is needed to investigate how such exposure might impact the behaviour and reproductive success of this and other butterfly species.

## Additional Information and Declarations

### Data Availability

The following information was supplied regarding data availability: Whitehorn, Penelope (2018): Data for_Larval exposure to the imidacloprid impacts adult size in Pieris brassicae.xlsx. figshare. Dataset. https://doi.org/10.6084/m9.figshare.5769360.

## References

[ref-1] Ahmad S, Ansari MS, Ahmad N (2013). Acute toxicity and sublethal effects of the neonicotinoid imidacloprid on the fitness of *Helicoverpa armigera* (Lepidoptera: Noctuidae). International Journal of Tropical Insect Science.

[ref-2] Baron GL, Jansen VAA, Brown MJF, Raine NE (2017b). Pesticide reduces bumblebee colony establishment and increases probability of population extinction. Nature Ecology & Evolution.

[ref-3] Baron GL, Raine NE, Brown MJF (2017). General and species-specific impacts of a neonicotinoid insecticide on the ovary development and feeding of wild bumblebee queens. Proceedings of the Royal Society B: Biological Sciences.

[ref-4] Bauerfeind SS, Fischer K (2008). Maternal body size as a morphological constraint on egg size and fecundity in butterflies. Basic and Applied Ecology.

[ref-5] Blacquière T, Smagghe G, Van Gestel CAM, Mommaerts V (2012). Neonicotinoids in bees: a review on concentrations, side-effects and risk assessment. Ecotoxicology.

[ref-6] Bonmatin JM, Moineau I, Charvet R, Collin ME, Fleche C, Bengsch ER, Lichtfourse E, Schwarzbauer J, Robert D (2005). Behavior of imidacloprid in fields. Toxicity for honey bees in environmental chemistry: green chemistry and pollutants in ecosytems.

[ref-7] Bonmatin J-M, Giorio C, Girolami V, Goulson D, Kreutzweiser DP, Krupke C, Liess M, Long E, Marzaro M, Mitchell EAD, Noome DA, Simon-Delso N. Tapparo, A (2015). Environmental fate and exposure; neonicotinoids and fipronil. Environmental Science & Pollution Research.

[ref-8] Botías C, David A, Hill E, Goulson D (2016). Contamination of wild plants near neonicotinoid seed-treated crops, and implications for non-target invertebrates. Science of the Total Environment.

[ref-9] Botías C, David A, Horwood J, Abdul-Sada A, Nicholls E, Hill E, Goulson D (2015). Neonicotinoid residues in wildflowers, a potential route of chronic exposure for bees. Environmental Science & Technology.

[ref-10] Brereton TM, Roy DB, Middlebrook I, Botham M, Warren M (2011). The development of butterfly indicators in the United Kingdom and assessments in 2010. Journal of Insect Conservation.

[ref-11] Buchholz A, Nauen R (2002). Translocation and translaminar bioavailability of two neonicotinoid insecticides after foliar application to cabbage and cotton. Pest Management Science.

[ref-12] David A, Botías C, Abdul-Sada A, Nicholls E, Rotheray EL, Hill EM, Goulson D (2016). Widespread contamination of wildflower and bee-collected pollen with complex mixtures of neonicotinoids and fungicides commonly applied to crops. Environment International.

[ref-13] Feltham H, Park K, Goulson D (2014). Field realistic doses of pesticide imidacloprid reduce bumblebee pollen foraging efficiency. Ecotoxicology.

[ref-14] Feltwell J (1982). Large white butterfly : the biology, biochemistry and physiology of pieris brassicae.

[ref-15] Forister ML, Cousens B, Harrison JG, Anderson K, Thorne JH, Waetjen D, Nice CC, De Parsia M, Hladik ML, Meese R, Van Vliet H (2016). Increasing neonicotinoid use and the declining butterfly fauna of lowland California. Biology Letters.

[ref-16] Gage MJG (1994). Associations between body size, mating pattern, testis size and sperm lengths across butterflies. Proceedings of the Royal Society B: Biological Sciences.

[ref-17] Gilburn AS, Bunnefeld N, Wilson JM, Botham MS, Brereton TM, Fox R, Goulson D (2015). Are neonicotinoid insecticides driving declines of widespread butterflies?. PeerJ.

[ref-18] Gill RJ, Ramos-Rodriguez O, Raine NE (2012). Combined pesticide exposure severely affects individual- and colony-level traits in bees. Nature.

[ref-19] Girolami V, Marzaro M, Vivan L, Mazzon L, Giorio C, Marton D, Tapparo A (2013). Aerial powdering of bees inside mobile cages and the extent of neonicotinoid cloud surrounding corn drillers. Journal of Applied Entomology.

[ref-20] Goulson D (2013). An overview of the environmental risks posed by neonicotinoid insecticides. Journal of Applied Ecology.

[ref-21] Gupta S, Gajbhiye VT, Gupta RK (2008). Soil dissipation and leaching behavior of a neonicotinoid insecticide thiamethoxam. Bulletin of Environmental Contamination and Toxicology.

[ref-22] Henry M, Béguin M, Requier F, Rollin O, Odoux J, Aupinel P, Aptel J, Tchamitchian S, Decourtye A (2012). A common pesticide decreases foraging success and survival in honey bees. Science.

[ref-23] Higginson AD, Delf J, Ruxton GD, Speed MP (2011). Growth and reproductive costs of larval defence in the aposematic lepidopteran *Pieris brassicae*. Journal of Animal Ecology.

[ref-24] Jeschke P, Nauen R, Schindler M, Elbert A (2011). Overview of the status and global strategy for neonicotinoids. Journal of Agricultural and Food Chemistry.

[ref-25] Jones A, Harrington P, Turnbull G (2014). Neonicotinoid concentrations in arable soils after seed treatment applications in preceding years. Pest Management Science.

[ref-26] Krupke CH, Hunt GJ, Eitzer BD, Andino G, Given K (2012). Multiple routes of pesticide exposure for honey bees living near agricultural fields. PLOS ONE.

[ref-27] Kurwadkar S, Wheat R, McGahan DG, Mitchell F (2014). Evaluation of leaching potential of three systemic neonicotinoid insecticides in vineyard soil. Journal of Contaminant Hydrology.

[ref-28] Limay-Rios V, Forero LG, Xue Y, Smith J, Baute T, Schaafsma A (2016). Neonicotinoid insecticide residues in soil dust and associated parent soil in fields with a history of seed treatment use on crops in southwestern Ontario. Environmental Toxicology and Chemistry.

[ref-29] Panizzi AR, Parra JR (2012). Insect bioecology and nutrition for integrated pest management.

[ref-30] Pecenka JR, Lundgren JG (2015). Non-target effects of clothianidin on monarch butterflies. The Science of Nature.

[ref-31] Pisa LW, Amaral-Rogers V, Belzunces LP, Bonmatin JM, Downs CA, Goulson D, Kreutzweiser DP, Krupke C, Liess M, McField M, Morrissey CA, Noome DA, Settele J, Simon-Delso N, Stark JD, Van der Sluijs JP, Van Dyck H, Wiemers M (2015). Effects of neonicotinoids and fipronil on non-target invertebrates. Environmental Science and Pollution Research.

[ref-32] R Core Team (2017). https://www.R-project.org/.

[ref-33] Roy DB, Rothery P, Moss D, Pollard E, Thomas JA (2001). Butterfly numbers and weather: predicting historical trends in abundance and the future effects of climate change. Journal of Animal Ecology.

[ref-34] Rundlöf M, Andersson GK, Bommarco R, Fries I, Hederström V, Herbertsson L, Jonsson O, Klatt BK, Pedersen TR, Yourstone J, Smith HG (2015). Seed coating with a neonicotinoid insecticide negatively affects wild bees. Nature.

[ref-35] Sánchez-Bayo F, Yamashita H, Osaka R, Yoneda M, Goka K (2007). Ecological effects of imidacloprid on arthropod communities in and around a vegetable crop. Journal of Environmental Science and Health, Part B.

[ref-36] Shirai Y (1995). Longevity, flight ability and reproductive performance of the diamondback moth, *Plutella xylostella* (L.) (Lepidoptera: Yponomeutidae), related to adult body size. Researches on Population Ecology.

[ref-37] Stanley DA, Smith KE, Raine NE (2015). Bumblebee learning and memory is impaired by chronic exposure to a neonicotinoid pesticide. Scientific Reports.

[ref-38] Stokstad E (2013). Pesticides under fire for risks to pollinators. Science.

[ref-39] Sur R, Stork A (2003). Uptake, translocation and metabolism of imidacloprid in plants. Bulletin of Insectology.

[ref-40] Van der Sluijs JP, Amaral-Rogers V, Belzunces LP, Bijleveld van Lexmond MFIJ, Bonmatin J-M, Chagnon M, Downs CA, Furlan L, Gibbons DW, Giorio C, Girolami V, Goulson D, Kreutzweiser DP, Krupke C, Liess M, Long E, McField M, Mineau P, Mitchell EAD, Morrissey CA, Noome DA, Pisa L, Settele J, Simon-Delso N, Stark JD, Tapparo A, Van Dyck H, Van Praagh J, Whitehorn PR, Wiemers M (2015). Conclusions of the Worldwide Integrated Assessment on the risks of neonicotinoids and fipronil to biodiversity and ecosystem functioning. Environmental Science and Pollution Research.

[ref-41] Waldbauer GP (1968). The consumption and utilization of food by insects. Advances in Insect Physiology.

[ref-42] Whitehorn PR, O’Connor S, Wackers F, Goulson D (2012). Neonicotinoid pesticide reduces bumble bee colony growth and queen production. Science.

[ref-43] Woodcock BA, Isaac NJ, Bullock JM, Roy DB, Garthwaite DG, Crowe A, Pywell RF (2016). Impacts of neonicotinoid use on long-term population changes in wild bees in England. Nature Communications.

